# Offspring reaction norms shaped by parental environment: interaction between within- and trans-generational plasticity of inducible defenses

**DOI:** 10.1186/s12862-016-0795-9

**Published:** 2016-10-12

**Authors:** Emilien Luquet, Juliette Tariel

**Affiliations:** CNRS UMR 5023 Ecologie des Hydrosystèmes Naturels et Anthropisés, Université Claude Bernard Lyon1, Université de Lyon, 43 Bd du 11 Novembre 1918, 69622 Villeurbanne cedex, France

**Keywords:** Phenotypic plasticity, Transgenerational plasticity, Inducible defenses, Predator-prey interactions, Reaction norm, Physa acuta

## Abstract

**Background:**

Within-generational plasticity (WGP) and transgenerational plasticity (TGP) are mechanisms allowing rapid adaptive responses to fluctuating environments without genetic change. These forms of plasticity have often been viewed as independent processes. Recent evidence suggests that WGP is altered by the environmental conditions experienced by previous generations (i.e., TGP). In the context of inducible defenses, one of the most studied cases of plasticity, the WGP x TGP interaction has been poorly investigated.

**Results:**

We provide evidence that TGP can alter the reaction norms of inducible defenses in a freshwater snail. The WGP x TGP interaction patterns are trait-specific and lead to decreased slope of reaction norms (behaviour and shell thickness). Offspring from induced parents showed a higher predator avoidance behaviour and a thicker shell than snails from non-induced parents in no predator-cue environment while they reached similar defenses in predator-cue environment. The WGP x TGP interaction further lead to a switch from a plastic towards a constitutive expression of defenses for shell dimensions (flat reaction norm).

**Conclusions:**

WGP-alteration by TGP may shape the adaptive responses to environmental change and then has a substantial importance to understand the evolution of plasticity.

**Electronic supplementary material:**

The online version of this article (doi:10.1186/s12862-016-0795-9) contains supplementary material, which is available to authorized users.

## Background

Phenotypic adaptation to fluctuating environments can occur through genetic evolution in response to natural selection over generations, or expression of traits within a generation in response to environmental cues (within-generational phenotypic plasticity, WGP). Furthermore, non-genetic inheritance of phenotypic responses induced by a variety of biotic and abiotic stresses [1] occurs in many organisms [2]. Transgenerational plasticity (TGP) is a change in offspring phenotype that is cued by an environmental signal in the parental generation (and possibly the previous ancestors) without involving genetic change in offspring [3]. It can occur through environmental influence on maternal (or more generally, parental) effects, whereby the phenotype of an offspring depends on the phenotype of its parents, regardless of their genotype [4–7]. Mecanistically, it may be mediated by parental care (as often assumed for parental effects), or by any form of epigenetic inheritance not involving changes in the DNA sequence, including DNA methylation marks, histone protein modifications and small RNA molecules [8–10].

WGP and more recently TGP have been highlighted as mechanisms allowing rapid adaptive responses to environmental change [3, 5, 11–13]. Nevertheless, the acquisition of the adaptive phenotype via WGP may be delayed by the lack of offspring sensory organs that can detect reliable cues early in development, and by the time required for major developmental changes [14, 15]. TGP may allow overcoming such developmental constraints on the timing of plastic responses [16]. By producing plastically induced offspring, parents pre-condition them to a given environment, improving their survival early in life before they detect the environmental cues [4, 5, 17]. TGP may also allow acquisition of more accurate information about the average environment, by integration over a longer duration [18].

**Fig. 1:**
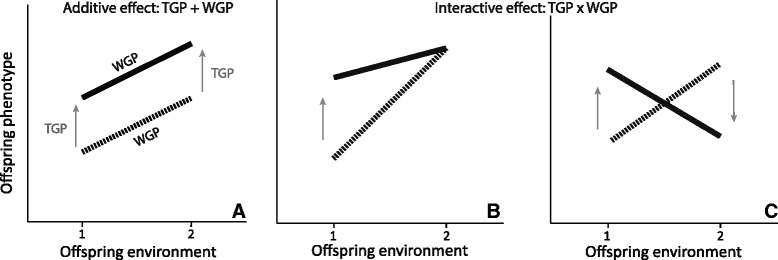
Distinction between within-generational plasticity (WGP) and transgenerational plasticity (TGP), and their additive or interactive effects. Additive effect: (**a**) offspring phenotype is independently affected by offspring environment (WGP) and parental environment (TGP). Interactive effect: (**b**) the parental environment only alters the slope of the offspring reaction norm or (**c**) the parental environment alters the offspring reaction norm direction, producing an opposite response for a same offspring environment. 1 et 2 are two disctinct environments where the phenotype of the offspring is measured. The black and dashed lines represent offspring reaction norms for two different parental environments

Thus, WGP and TGP both play a key role in adaptive responses to environmental changes. However, they are expected to evolve in slighlty different contexts [19]. WGP is selected when environments are spatially and/or temporally heterogeneous [20], costs of plasticity are low [15, 21] and cues reflect the state of the environment where selection operates on the phenotype [22–25]. In contrast, TGP is favoured when environmental (temporal) heterogeneity across generations and costs of obtaining information and responding are low, and when parental environment is a good proxy of offspring environment [1, 10, 14, 18, 26, 27]. These forms of plasticity have often been viewed as separate processes, occuring independently from each other and thus contributing additively to the offspring phenotype (additive effect; Fig. 1a; Beaman et al. [28]). The majority of studies have only investigated how TGP can change mean trait values. Yet, recent evidence suggested that TGP can alter the WGP reaction norm (i.e., the full set of phenotypic responses to an environmental variable within a generation; Salinas et al. [29] for a review). This interaction between TGP and WGP can have substantial importance to understand the evolution of plasticity, because it can shape the adaptive strategies in response to environmental change. WGP x TGP interaction may allow (i) to reach the adaptive phenotype faster and/or with a lower cost (Fig. 1b) or (ii) to change the adaptive phenotype according to the parental environment (change in direction of the offspring reaction norms; Fig. 1c). A recent theory suggests that the strongest transgenerational effects occur for traits that experience very strong selection and for which WGP is severely constrained [19]. Such a balance between WGP and TGP can shape the adaptive strategies in response to environmental change.

Inducible defenses are one of the most studied cases of WGP. A great variety of plants and animals produce ennemy-specific phenotypes by detecting enemy cues released in the environment [3, 30]. There are numerous examples of morphological defenses induced by predator kairomones (cues) in animals. The most famous one is the expression of neckteeth in some Daphnia species, that is also the example used to define the terms of reaction norms [31]. Inducible defenses are an example of adaptive phenotypic plasticity improving fitness in presence of ennemies while avoiding potential costs associated with defense production when it is not needed [30]. Such evidence of phenotypic benefits and costs have been particularly demonstrated in predator-prey systems for inducible morphology, life-history and behaviour defenses in animals [32–37].

Parental exposure to predator cues can significantly alter the offspring phenotype (TGP), such as modifications in various life-history traits [38–40] or induction of defensive morphologies [16, 41, 42] or behaviour [43–46]. TGP of defenses may be highly advantageous and positively selected in nature [3, 12, 16], and may thus be an important component of predator-prey interaction. Surprisingly, very few studies investigated the TGP on the offspring reaction norms of defensive traits themselves (e.g., Agrawal et al., [16]; Beaty et al., [42]). The pioneering study of Agrawal et al. [16] investigated the transmission of the anti-predator morph in *Daphnia cucullata* across several generations and showed that individuals with induced neckteeth produced offspring with a typical predator-induced morph. However, there was no change in offspring reaction norms and their results thus demonstrated that WGP and TGP contributed additively to the offspring phenotype. Beaty et al. [42] found a similar additive contribution for one defense trait while other traits were exclusively affected by TGP or WGP in a freshwater snail. Changes in offspring reaction norms via TGP (interactive effect) are however well-known in other contexts [28, 29] but, to our knowledge, they have never been demonstrated for inducible defense traits.

In this study, we conducted an experimental two-generation study to investigate how WGP and TGP can interact to shape the inducible defenses of the freshwater snail *Physa acuta* (behaviour and shell morphology) in response to predator cues. The hermaphroditic *Physa* gastropods have been used in numerous works studying inducible defenses (e.g., DeWitt [37]; DeWitt et al., [47]; Auld & Relyea [48]; Gustafson et al., [49]; Auld & Houser [50]; Beaty et al., [42]). Modifications of shell morphology and behaviour induced by predator cues are thus well-described (shell-crushing resistant: e.g., thicker shell, rotund shell shape and predator avoidance behaviour). In addition, the short generation time of this species allows to conduct an experiment across multiple generations. We compared the offspring reaction norms of inducible defenses (predator avoidance and shell-crushing resistant morphology) in response to parental and offspring environments (control and predator cues). We expected that parental environment can generate (i) an additive effect leading to an increase of maximal defenses in predator-cue offspring environment (higher proportion of predator avoidance, thicker shell, more rotund shell shape; Fig. 1a) and/or (ii) a WGP x TGP interaction, with a change in the slope (but not necessarily direction) of offspring reaction norms (Fig. 1b).

## Methods

### Animal collection and experimental design

Adult *P. acuta* snails were collected (*n* = 150) in March 2015 in a lentic backwater of the Rhône river (45.80° N, 4.92° E) in Lyon, France. Predators (fish and crayfish) are present in this natural environment. We pooled all snails overnight in a 10 L-aquarium to ensure that offspring result from outcrossing (*P. acuta* is a preferential outcrosser; Jarne et al. [51]). Then, we individually isolated all snails in 70 mL plastic boxes filled with reconstituted water (2.4 g NaHCO_3_, 3 g CaSO_4_, 1.5 g MgSO_4_, 0.1 g KCl to 25 L deionized water; pH 6.83; [Ca2+] = 28 mg/L) in a 25 °C experimental room with 12 h light–dark photoperiod. After 24 h, we haphazardly selected 15 of these wild-caught (G0) snails that had laid a first egg capsule each. These 15 egg capsules (hereafter called “families”) developed until hatching (~7 days) and constituted the parental generation (G1). Two days after hatching, we haphazardly sampled 12 siblings per family and split them into two environments: six snails remained in a no-predator environment while 6 others were moved in a predator-cue environment (*n* total = 180 individuals). These G1 snails were reared in 70 mL plastic boxes with a constant density of six sibling/box. At 28-days old, they were isolated in the same type of plastic boxes. Boxes were closed to prevent snail escape. 7 G1 snails died during the experiment. At 35-days old, we generated the second generation. Predator cues were obtained by individually rearing crayfishes (*Procambarus clarkii*) in 4 L reconstituted water and feeding every 3 days with ~ 200 mg of *P. acuta* [48]. Twice a week, at each water renewal, the crayfish-conditioned water was used for the predator-cue treatment. Snails in the control treatment were always reared only in reconstituted water. Water and food (*ad libitum*, chopped and boiled lettuce) were renewed at the same frequency for all experimental snails.

To generate the offspring snail generation (G2), we formed six copulation groups per treatment. Each copulation group included 15 G1 adult snails (one individual per G1 family per treatment). Each group was placed for 24 h in a 5 L aquarium and thereafter individuals were isolated. After 24 h, we haphazardly selected 17 G1 snails from each treatment that had laid eggs (i.e., 17 families per parental treatment). We then followed the same protocol as previously to rear G2 snails in no-predator or predator-cue environments (*n* total = 408). 33 G2 snails died during the experiment. We measured the phenotypic traits at 49-days old. Individuals of the G2 generation consequently corresponded to one of the four life-history lineages: (i) control parents - > control offspring, (ii) control - > predator-cues, (iii) predator-cues - > control and (iv) predator-cues - > predator-cues (Additional file 1).

### Behavioural assay and morphology measurements

To investigate TGP of anti-predator defenses, we estimated, on both G1 and G2, snails predator avoidance behaviour and measured six morphological traits (total weight, shell thickness, shell length, shell width, aperture length and aperture width) known to be influenced by predator cues and involded in adaptive anti-predator responses [37, 47, 48]. Snail behavioural response was recorded in their rearing boxes with predator cues present or absent according to the treatment, 24 h after a water change, 1 week before the morphology measurements. Predator avoidance behaviour was estimated by the proportion of snails crawling to the water surface or out of the water (called crawling-out behaviour hereafter). Snails were photographed and the software ImageJ (http://imagej.nih.gov/ij/) was used to measure the maximum lengths (shell length, shell width, aperture length and aperture width). The shell shape was assessed by calculating the shell length / shell width ratio. Shell thickness was measured with a numerical caliper at the nearest 0.01 mm. Snails were weighed with an electronic scale at the nearest 0.001 mg.

### Statistical analyses

The effect of predator cues on predator avoidance behaviour was analyzed using generalized linear mixed models assuming a binomial distribution (logit link function). The effect of predator cues on morphology was analyzed using linear mixed models with restricted maximum likelihood estimation and Kenward and Roger’s approximation for degrees of freedom. Because all traits were strongly correlated with weight, we used analyses of covariance (ANCOVA) to control weight effect (log transformation) on the dependent variables, except for the shell length / shell width ratio (no significant correlation with weight). Interactions between the weight covariate and other fixed effects were removed when non-significant.

Parental (E1) and offspring (E2) environments (control or predator-cue) and their interaction were considered as fixed effects, and family was considered as a random intercept effect. When interactions with the weight covariate were significant (shell length and shell width), we splitted the weight covariate into two groups around the median (pre- and post- weight median) and then analyzed the effects of parental and offspring environments in each case. The results on aperture dimensions were all non-significant and were then not shown. The results on G1 generation are shown in supplementary material (Additional file 2). Generalized mixed models were done in R 3.2.1 (R Core Team 2015) with the glmmPQL function (MASS package) and linear mixed models were done in JMP 10 (SAS Institute, NC).

## Results

**Fig. 2:**
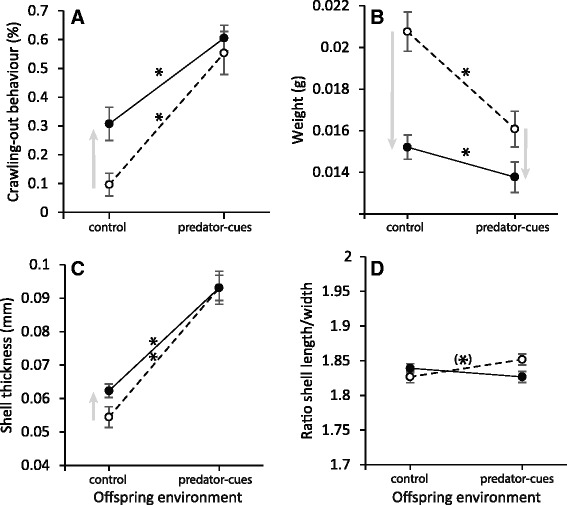
Reaction norms of offspring phenotype (G2) according to parental (E1) and offspring (E2) environments for **a** crawling-out behaviour, **b** weight, **c** shell thickness and **d** ratio shell length / shell width. *White circle* and *dashed line* show reaction norms of offspring from control parental environment. *Black circle* and *solid line* show reaction norms of offspring from predator-cue parental environment. Significant TGP and WGP are showed by *grey arrows* and asterisk (*) respectively (see Table 1 & Additional file 3)

An interaction of Parental-by-Offspring environments affected crawling-out behaviour (GLMM, Parental env.: t_35_ = 2.928, *p* =0.006, Offspring env.: t_35_ = 5.5185, *p* < 0.001; Parental x Offspring env.: t_35_ = −2.177, *p* = 0.036; Fig. 2a) and shell thickness (Fig. 2c; Table 1), demonstrating that parental environment causes a shift in the slope of offspring reaction norms. Surprisingly, this interaction was sometimes antagonistic, such that exposing the parents to the predator cue reduces the response of their offspring to this cue. For instance, the increase in crawling-out behaviour and shell thickness in environment with predator cues was higher for offspring from control parental environment (45 and 71 %, respectively) than those from predator-cue parental environment (30 and 49 %, respectively). Regarding the direct effect of parental environment on offspring phenotype (TGP), offspring from predator-cue parental environment had a higher proportion of crawling-out behaviour and a thicker shell in control environment, but showed similar behaviour and thickness than offspring from control parental environment in predator cues environment (Fig. 2; Additional file 3 for contrast statistics). Parental and offspring environments additively affected weight (Fig. 2b; Table 1). Both parental and offspring environments reduced the weight in presence of predator-cues.

**Table 1:** Results of linear mixed models analyses of (co)variance for the offspring generation (G2)

Weight		Estimates (SE)	Numdf, Dendf	F	P
	Parental env. (E1)	−0.0020 (0.0008)	1, 34.31	6.95	0.0125*
	Offspring env. (E2)	−0.0014 (0.0004)	1, 336.73	15.02	0.0001*
	E1 x E2	0.0007 (0.0004)	1, 336.73	3.38	0.0667
	*Random effect*	*Var*	*SE*	*Z*	*P*
	*Family*	*0.0000169*	*0.00000536*	*3.12*	0.0008*
Shell thickness		Estimates (SE)	Numdf, Dendf	F	P
	Weight (W)	0.0333 (0.0034)	1, 340.63	98.21	<0.0001*
	Parental env. (E1)	0.0053 (0.0023)	1, 37.49	5.24	0.0278*
	Offspring env. (E2)	0.0204 (0.0017)	1, 343.66	149.82	<0.0001*
	E1 x E2	−0.0035 (0.0016)	1, 341.77	4.39	0.0368*
	*Random effect*	*Var*	*SE*	*Z*	*P*
	*Family*	*0.0000955*	*0.0000461*	*2.07*	0.019*
Shell length		Estimates (SE)	Numdf, Dendf	F	P
	Weight (W)	1.7214 (0.0206)	1, 352.56	6999.59	<0.0001*
	Parental env. (E1)	−0.0311 (0.0161)	1, 37.23	3.72	0.0613
	Offspring env. (E2)	−0.0299 (0.0104)	1, 341.09	8.27	0.0043*
	W x E1	−0.0572 (0.0206)	1, 352.56	7.74	0.0057*
	W x E2	−0.0604 (0.0194)	1, 360.90	9.65	0.0020*
	E1 x E2	0.0035 (0.0104)	1, 341.09	0.11	0.7347
	W x E1 X E2	0.0336 (0.0194)	1, 360.90	2.99	0.0845
	*Random effect*	*Var*	*SE*	*Z*	*P*
	*Family*	*0.00553*	*0.002214*	*2.50*	0.0062*
Shell width		Estimates (SE)	Numdf, Dendf	F	P
	Weight (W)	0.9254 (0.0121)	1, 347.72	5867.74	<0.0001*
	Parental env. (E1)	−0.0150 (0.0090)	1, 34.82	2.76	0.1054
	Offspring env. (E2)	−0.0254 (0.0059)	1, 340.22	18.02	<0.0001*
	W x E1	0.0136 (0.0061)	1, 341.69	4.91	0.0274*
	W x E2	−0.0226 (0.0120)	1, 349.57	3.56	0.0600
	E1 x E2	−0.0438 (0.0114)	1, 362.95	14.64	0.0002*
	*Random effect*	*Var*	*SE*	*Z*	*P*
	*Family*	*0.0016328*	*0.0007181*	*2.27*	0.0114*
Ratio shell length / width		Estimates (SE)	Numdf, Dendf	F	P
	Parental env. (E1)	−0.0034 (0.0058)	1, 32.95	0.34	0.5635
	Offspring env. (E2)	0.0024 (0.0040)	1, 339.80	0.35	0.5563
	E1 x E2	−0.0087 (0.0040)	1, 339.80	4.66	0.0316*
	*Random effect*	*Var*	*SE*	*Z*	*P*
	*Family*	*0.0006571*	*0.0003111*	*2.11*	0.0173*

Italic values show random effects

*symbol indicates *P* < 0.05. Side-by-side comparisons (contrast method) are showed in Additional file 3

**Fig. 3:**
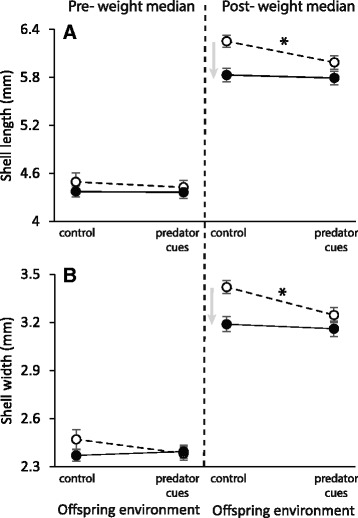
Reaction norms of offspring according to parental (E1) and offspring (E2) environments around the weight median for **a**) shell length and **b**) shell width. *White circle* and *dashed line* show reaction norms of offspring from control parental environment. *Black circle* and *solid line* show reaction norms of offspring from predator-cue parental environment. Significant TGP and WGP are showed by *grey arrows* and asterisk (*) respectively (Additional file 3). The overall relationships between shell length and shell width and weight of offspring phenotype (G2) according to parental and offspring environments are showed in Additional file 4

Both parental and offspring environment effects on shell length and shell width interacted with weight (Fig. 3; Table 1). The slice tests revealed that a significant Parental-by-Offspring environment interaction affected shell length and shell width above the weight median (F_1, 167.7_ = 4.49, *p* = 0.0355 and F_1, 165.4_ = 8.95, *p* = 0.0032 respectively; Fig. 3; Additional file 4) but was not significant below the weight median (Fig. 3; Additional file 4). While the heaviest offspring from control parental environment showed a decrease in shell length and shell width with predator cues (Fig. 3), the heaviest offspring from predator-cue parental environment were globally shorter and narrower, and did not show a decrease in shell length and shell width with predator cues (flat reaction norms; Fig. 3; Additional file 3 for contrast statistics).

The shell length / width ratio was affected by a significant interaction between parental and offspring environments (Table 1). Offspring from control parental environment showed a marginal increase with predator cues while offspring from predator-cue parental environment were not affected by predator cues (Fig. 2d; Additional file 3 for contrast statistics). The family random effect was significant for every traits (Table 1).

## Discussion

Our study showed TGP of defensive traits in *P. acuta*. Parents exposed to predator cues produced offspring with higher anti-predator defenses (predator avoidance behaviour and crush-resistant shell shape). More interestingly, we demonstrated that TGP can further alter the reaction norms of inducible defenses. Parental and offspring environments interacted to shape the reaction norms of inducible defenses in offspring. The most striking result was the switch from a plastic towards a constitutive expression of smaller shell dimensions (length and width) in offspring from induced parents. In other words plasticity in offspring was lost in response to the predator-cue environment of parents.

G1 snails in predator-cue environment exhibited anti-predator defenses (Additional file 2) as observed in numerous studies [42, 48, 49, 52]. Such parental phenotypes induced by environment can be inherited in offspring. Some examples exist both in plants and animals [53, 54]. TGP is predicted to be selected when the parental environment reliably predicts the offspring environment [14, 27, 55]. This is particularly expected in species with a short-generation time and a low dispersal like *P. acuta* because offspring are likely to live in the same environment than their parents [56]. Our study showed TGP for weight and all defensive traits of offspring G2 snails. Offspring from induced parents were lighter with significantly higher crawling-out behaviour, thicker shells and smaller shell dimensions in control environment. Although we did not measured fitness directly, several studies on gastropods showed that a crawling-out behaviour, a thicker shell, smaller shell dimensions and a rounded shape increase survival in crayfish-predation risk environment and are then considered as an adaptive response [37, 48]. Such TGP may be adaptive because it programs the defenses of offspring to the potential predation risk in the offspring environment, and importantly is in the same direction as WGP. However, the decrease in weight in offspring from induced parents may also be a transgenerational cost, and resulting from a higher investment of parents in defenses than in progeny. Predation risk is indeed known to affect the decision making of prey between predation avoidance and energy intake, and to increase the maternal stress acting on offspring development [50, 57, 58]. These results further suggest that offspring cannot totally overcome the parental environment effect because TGP was detected late in offspring development. The persistence of parental environment effect in offspring, even in the absence of predator cues, may lead to mismatched strategies for parents and offspring (i.e., parent-offspring conflicts) and thus to a maladptive TGP if the environment varies across generations [14].

Our study also revealed that TGP can alter the reaction norms of inducible defenses. The slope of offspring reaction norms for crawling-out behaviour, shell thickness and shell dimensions depended on the interaction between parental and offspring environments. Interestingly, offspring from both non-induced and induced parents reached overall a similar protection (crawling-out behaviour, shell thickness and shell dimensions) in predator-cue environment although reaction norms were different. This contrasts with the result on weight and a previous study on transgenerational induction of defense where Daphnia from induced parents increased the maximal helmet length in predator cue environment (i.e., additive effect; Agrawal et al. [16]). Our results rather suggest that the snail defensive traits may be constrained to maximal values in the experimental environment, which is probably not true for the weight because of *ad libitum* food. The anti-predator phenotype can be limited by the current environmental conditions because of production costs [15]. Such costs have already been demonstrated for induced-shell morphology defenses in *Physa sp.* [37]. Another possibility is that shell morphology was constrained by abiotic nutrients availability in environment. Bukowski and Auld (2014) [59] indeed demonstrated that calcium availability strongly affected the predator-induced shell morphology. Thus, constraints acting on inducible defenses can limit the evolution of additive effect (i.e., an increase of phenotypic maximal value) and generate WGP x TGP interactions.

Two different patterns of WGP x TGP interaction occured depending on the defensive traits. Some traits responded more strongly to the parental than to the offspring environment. Crawling-out behaviour and shell thickness increased in offspring predator-cue environment for both parental environments while shell dimensions (length and width) were only affected by offspring predator-cues for non-induced parental environment. Predator-induced TGP on crawling-out behaviour and shell thickness responses decreased the slope of the reaction norms. The parental environment induced intermediate defenses in offspring, which allows for offspring to keep a flexibility to react to the current environmental conditions, thus limiting the risk of maladaptive TGP. Surprisingly, TGP on shell dimensions led to constitutive defenses for offspring from induced parents (flat reaction norms). In this case, the maximum possible phenotypic response is already reached in response to parental environment (TGP), regardless of offspring environment, so no more WGP is required. Plasticity to the offspring environment is thus by-passed by the response to parental environment. The canalization of defenses in pre-conditioned offspring would immediately allow for the best protection against potential predation risk, and could be a cost-saving strategy, but can lead to maladaptation if the environmental condition varies [3]. Such conflicting impacts of parental environment on reaction norms can result from a balance between WGP and TGP. A recent theoretical study showed that the largest effects of parental environment occured for traits with a low or severely constrained plasticity [19]. TGP would be favored for the most constrained plastic traits because it would be the only means of adaptation to a fluctuating environment. Crawling-out behaviour and shell thickness were the traits that exhibit the higher WGP (45 and 71 % respectively) in our study (and in DeWitt et al. [47]; Auld & Relyea [48]) compared to shell dimensions (4 and 5 % for shell length and shell width respectively). The reaction norms for crawling-out behaviour and shell thickness were consistently the least affected by parental environment in our study. This is also consistent with Beaty et al. [42] study demonstrating that anti-predator behaviour was only affected by the current environment while crush resistance was only influenced by the parental one or shell size by both parental and offspring environments. The WGP of shell dimensions is probably more constrained by developmental processes than behaviour and shell thickness. High TGP on shell dimensions would allow to get a defensive shell morphology despite developmental constraints.

Some examples already showed that TGP can change the magnitude and even the direction of reaction norms (reviewed in Salinas et al. [29]). Here, we further demonstrated that TGP can involve a by-pass of WGP towards canalization of defenses in a subsequent offspring generation and without natural or artificial selection. In some cases, WGP can become reduced or lost, either from selection against costly developmental machinery underlying plasticity or because of relaxed selection when alternative environments are not frequently encountered [60]. Genetic assimilation is the complete loss of WGP, whereby an environmentally induced trait is selected to become constitutively expressed without the original environmental cue [25, 61, 62]. Our study suggests that TGP can favor the genetic assimilation of some defensive plastic traits when environment is stable. This result can have important implications for the evolution of inducible defenses. By influencing both phenotypic variance and offspring fitness, TGP can shape the course of genetic evolution in newly environmental conditions and accelerate the evolutionary response to predation risk [28, 55, 60, 63–65].

## Conclusions

Consequently, predator-induced TGP responses on inducible defenses themselves may allow for a rapid adaptive response to predation risk and initiate evolutionary changes. The key distinction from the standard model of evolution from genetic variation is that evolutionary significant phenotypic novelty can arise from environmental and/or non-genetic alterations of the genotype-to-phenotype map [60]. Some theoretical studies modelled the rate of evolution with the possibility of such non-genetic inheritance [1, 26] but it is still unclear how WGP x TGP interaction evolves [11, 13]. More theoretical and empirical studies on TGP and especially in the inducible defenses context are required to understand what determines which environments (past or current) shape the phenotype [42]. A dynamic view of defense induction according to parental environment would be further interesting to improve our understanding of adaptiveness of WGP x TGP interaction (e.g., faster building of anti-predator defense). Recently, Walsh et al. (Walsh et al. [66]) provided evidence that divergent ecological conditions in temporal variation of predation risk can select for WGP or TGP of life-history traits. However, they did not investigate how TGP alters WGP according to the ecological conditions. A next challenging question is to determine the genetic and non-genetic parts of inducible defense heritability [67]. The persistent effect of family on every measured traits indicated that both genetic variation and/or maternal effects occured. Furthermore, Dillon & Jacquemin (2015) [68] showed a high heritability of shell morphological traits in *Physa*. An intensive knowledge is available on maternal effects and transmission of various factors in ovocytes [4, 5]. However, environmental stressors like predators can potentially induce phenotypic change than span multiple generations [39]. Non-genetic inheritance of inducible defenses on more than two generations and underlying molecular mechanisms (e.g., epigenetic variation) deserve to be explored [1, 9, 17].

## Additional files

Additional file 1: Experimental design for evaluating WGP x TGP interaction. (DOC 60 kb)
Additional file 2: Results on G1 generation (DOC 64 kb)
Additional file 3: Contrast method on the estimates of models for shell thickness, shell length, shell width and ratio shell length / shell width in the G2 generation (see Table 1 for analyses of covariance). (DOC 49 kb)
Additional file 4: Relationships between A) shell length or B) shell width and weight of offspring phenotype (G2) according to parental (E1) and offspring (E2) environments (DOC 112 kb)
Additional file 5: Raw dataset used in this study. (XLSX 37 kb)
