# Supplementary material for: Offspring reaction norms shaped by parental environment: interaction between within- and trans-generational plasticity of inducible defenses
Source: BMC Evol Biol. 2016 Oct 12;16:209. doi: 10.1186/s12862-016-0795-9 (PMC5062831; doi:10.1186/s12862-016-0795-9)
Supplement: Additional file 1: — Experimental design for evaluating WGP x TGP interaction. (DOC 60 kb) [file 12862_2016_795_MOESM1_ESM.doc]

**Additional file 1.** Experimental design for evaluating WGP x TGP interaction.


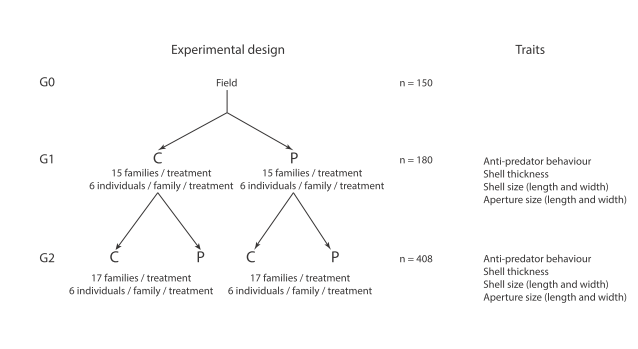


**“**C” – control (no predator cues) treatment. “P” – predator-cue treatment. G0 is the founder generation (adult snails from the wild). G1 and G2 are the parental and offspring generations respectively**.**
