# Supplementary material for: Offspring reaction norms shaped by parental environment: interaction between within- and trans-generational plasticity of inducible defenses
Source: BMC Evol Biol. 2016 Oct 12;16:209. doi: 10.1186/s12862-016-0795-9 (PMC5062831; doi:10.1186/s12862-016-0795-9)
Supplement: Additional file 2: — Results on G1 generation (DOC 64 kb) [file 12862_2016_795_MOESM2_ESM.doc]

**Additional file 2.** Results on G1 generation

*Statistical analysis –* Environment (control or predator-cue) was considered as a fixed factor, and family was considered as a random effect.

*Results -* Predator cues significantly affected crawling-out behaviour, weight, shell thickness and shell size (see Table below). A significantly higher proportion of snails showed a crawling-out behaviour in predator-cue environment compared to control environment (GLMM, t_14_ = 5.42, p < 0.01; see Table below). Predator cues induced a 71% increase in weight (F_1, 156.2_ = 119.67, p < 0.001; see Table below) and a 55% thicker shell (F_1, 155.1_ = 16.22, p < 0.001; see Table below). The effects of predator cue on shell length and width significantly depended on weight (significant weight x environment interaction: F_1, 158_ = 4.39, p = 0.0378 and F_1, 155.3_ = 4.91, p = 0.0282 respectively; see Table below). These traits increased with predator cues (see Table below), but after adjusting for weight, predator induction resulted in relatively shorter shell length and shell width (see Table below). We found no effect of predation cues on shell length / width ratio (F_1, 158.2_ = 0.97, p = 0.3268; see Table below).

*Discussion -*Surprisingly, G2 snails were lighter in the predator-cue environment while their parents (G1) were heavier. Such a difference in direction of reaction norms between G1 and G2 individuals suggests that different transgenerational effects can affect the G1 and G2 snail phenotypes. Because parents of G1 snails (G0) have been sampled from the wild, G1 phenotypes were shaped by both the parental wild environment and the current experimental environment. Thus, the G1 reaction norms may not be mainly explained by a crayfish-cue induction and probably result from strong multiple stress effects.

Trait means (± standard error) for the parental generation (G1).

| Trait means |  | |  |  |  |  |
| --- | --- | --- | --- | --- | --- | --- |
|  | Environment | |  |  |  |  |
|  | Control | |  | Predator cues | |  |
| Traits | Mean(SE) | |  | Mean(SE) | |  |
| Crawling-out behaviour (%) | 17(0.05) | |  | 73(0.06) | |  |
| Weight (g) | 0.0144(0.0005) | |  | 0.0247(0.0008) | |  |
| Shell thickness (mm) | 0.09(0.003) | |  | 0.14(0.01) | |  |
| Shell length (mm) | 5.021(0.063) | |  | 5.888(0.077) | |  |
| Shell width (mm) | 2.779(0.035) | |  | 3.279(0.04) | |  |
| Ratio shell length/width | 1.807(0.003) | |  | 1.796(0.003) | |  |
|  |  |  |  |  |  |  |

Results of the linear mixed model analyses of (co)variance for the G1 generation.

| Weight |  | **Estimates (SE)** | **Numdf, Dendf** | **F** | **P** |
| --- | --- | --- | --- | --- | --- |
|  | Environment | 0.0052 (0.0005) | 1, 156.2 | 119.67 | <0.0001* |
|  | *Random effect* | ***Var*** | ***SE*** | ***Z*** | ***P*** |
|  | *Family* | *0.000000289* | *0.00000122* | *0.24* | *0.4064* |
|  |  |  |  |  |  |
| Shell thickness |  | **Estimates (SE)** | **Numdf, Dendf** | **F** | **P** |
|  | Weight | 0.0428 (0.0089) | 1, 157.3 | 22.82 | <0.0001* |
|  | Environment | 0.0147 (0.0036) | 1, 155.1 | 16.22 | <0.0001* |
|  | *Random effect* | ***Var*** | ***SE*** | ***Z*** | ***P*** |
|  | *Family* | *0.0000479* | *0.0000645* | *0.74* | *0.2288* |
|  |  |  |  |  |  |
| Shell length |  | **Estimates (SE)** | **Numdf, Dendf** | **F** | **P** |
|  | Weight | 1.8246 (0.0695) | 1, 155.6 | 688.65 | <0.0001* |
|  | Environment | -0.0619 (0.0284) | 1, 153.6 | 4.75 | 0.0309* |
|  | Weight x Env. | 0.1476 (0.0705) | 1, 158 | 4.39 | 0.0378* |
|  | *Random effect* | ***Var*** | ***SE*** | ***Z*** | ***P*** |
|  | *Family* | *0.0033674* | *0.0039169* | *0.86* | *0.1950* |
|  |  |  |  |  |  |
| Shell width |  | **Estimates (SE)** | **Numdf, Dendf** | **F** | **P** |
|  | Weight | 1.0233 (0.0367) | 1, 158.6 | 767.49 | <0.0001* |
|  | Environment | -0.0198 (0.0151) | 1, 156.1 | 1.73 | 0.1898 |
|  | Weight x Env. | 0.0825 (0.0372) | 1, 155.3 | 4.91 | 0.0282* |
|  | *Random effect* | ***Var*** | ***SE*** | ***Z*** | ***P*** |
|  | *Family* | *0.00003869* | *0.0008072* | *0.0479* | *0.48* |
|  |  |  |  |  |  |
| Ratio shell length / width |  | **Estimates (SE)** | **Numdf, Dendf** | **F** | **P** |
|  | Environment | -0.0075 (0.0076) | 1, 158.2 | 0.97 | 0.3268 |
|  | *Random effect* | ***Var*** | ***SE*** | ***Z*** | ***P*** |
|  | *Family* | *0.0005127* | *0.000517* | *0.99* | *0.1607* |

* symbol indicates P < 0.05.
