# Supplementary material for: Offspring reaction norms shaped by parental environment: interaction between within- and trans-generational plasticity of inducible defenses
Source: BMC Evol Biol. 2016 Oct 12;16:209. doi: 10.1186/s12862-016-0795-9 (PMC5062831; doi:10.1186/s12862-016-0795-9)
Supplement: Additional file 3: — Contrast method on the estimates of models for shell thickness, shell length, shell width and ratio shell length / shell width in the G2 generation (see Table 1 for analyses of covariance). (DOC 49 kb) [file 12862_2016_795_MOESM3_ESM.doc]

**Additional file 3.** Contrast method on the estimates of models for shell thickness, shell length, shell width and ratio shell length / shell width in the G2 generation (see Table 1 for analyses of covariance).

**Weight – Fig. 2B**

|  | Control-Control | Control-Predator | Predator-Control | Predator-Predator |
| --- | --- | --- | --- | --- |
| Control-Control | / | **t_336.19_ = -4.13**  **p < 0.0001** | **t_52.55_ = 3.16**  **p = 0.0026** | **t_53.15_ = 4.02**  **p = 0.0002** |
| Control-Predator | / | / | t_50.17_ = 0.70  p = 0.4868 | t_50.76_ = 1.58  p = 0.1194 |
| Predator-Control | / | / | / | t_337.23_ = 1.41  p = 0.1596 |

**Shell thickness – Fig. 2C**

|  | Control-Control | Control-Predator | Predator-Control | Predator-Predator |
| --- | --- | --- | --- | --- |
| Control-Control | / | **t_341.59_ = -10.32**  **p < 0.0001** | **t_84.45_ = -3.04**  **p = 0.0032** | **t_87.43_ = -8.73**  **p < 0.0001** |
| Control-Predator | / | / | **t_74.92_ = 5.41**  **p < 0.0001** | t_76.20_ = -0.67  p = 0.51 |
| Predator-Control | / | / | / | **t_343.79_ = -7.14**  **p < 0.0001** |

**Shell length (Upper weight median group) – Fig. 3A**

|  | Control-Control | Control-Predator | Predator-Control | Predator-Predator |
| --- | --- | --- | --- | --- |
| Control-Control | / | **t_167.08_ = 3.40**  **p = 0.0008** | **t_59.58_ = 1.99**  **p = 0.0502** | **t_72.98_ = 1.93**  **p = 0.0570** |
| Control-Predator | / | / | t_66.75_ = -0.49  p = 0.6251 | t_80.14_ = -0.41  p = 0.6834 |
| Predator-Control | / | / | / | T_169.05_ = 0.067  p = 0.9464 |

**Shell width (Upper weight median group) – Fig. 3B**

|  | Control-Control | Control-Predator | Predator-Control | Predator-Predator |
| --- | --- | --- | --- | --- |
| Control-Control | / | **t_164.75_ = 4.81**  **p < 0.0001** | **t_51.17_ = 2.084**  **p = 0.042** | **t_64.05_ = 2.04**  **p = 0.0454** |
| Control-Predator | / | / | T_57.98_ = -1.46  p = 0.1492 | t_71.10_ = -1.29  p = 0.1994 |
| Predator-Control | / | / | / | t_16.91_ = 0.10  p = 0.9189 |

**Ratio shell length / shell width – Fig. 2D**

|  | Control-Control | Control-Predator | Predator-Control | Predator-Predator |
| --- | --- | --- | --- | --- |
| Control-Control | / | **t_338.13_ = -2.28**  **p = 0.0233** | t_69.95_ = -1.00  p = 0.3186 | t_72.88_ = -0.26  p = 0.7980 |
| Control-Predator | / | / | t_62.27_ = 0.75  p = 0.4530 | t_63.41_ = 1.51  p = 0.1358 |
| Predator-Control | / | / | / | T_339.21_ = 0.95  p = 0.3415 |
