# Supplementary material for: Offspring reaction norms shaped by parental environment: interaction between within- and trans-generational plasticity of inducible defenses
Source: BMC Evol Biol. 2016 Oct 12;16:209. doi: 10.1186/s12862-016-0795-9 (PMC5062831; doi:10.1186/s12862-016-0795-9)
Supplement: Additional file 4: — Relationships between A) shell length or B) shell width and weight of offspring phenotype (G2) according to parental (E1) and offspring (E2) environments (DOC 112 kb) [file 12862_2016_795_MOESM4_ESM.doc]

**Additional file 4.** Relationships between A) shell length or B) shell width and weight of offspring phenotype (G2) according to parental (E1) and offspring (E2) environments


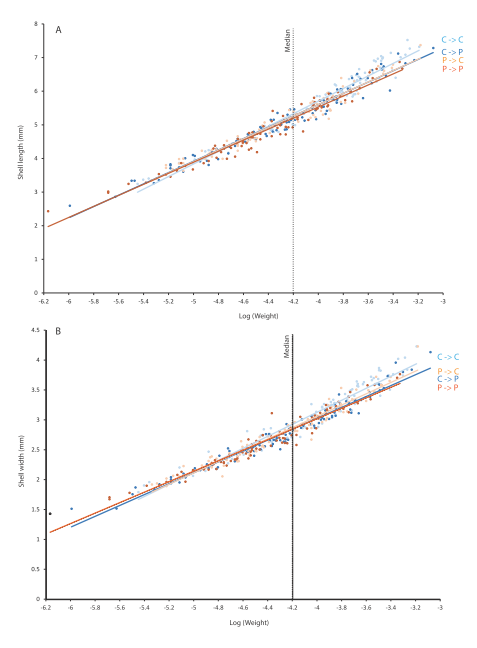


(C -> C: control-control; P -> C predator-control; C -> P: control-predator; P -> P: predator-predator).

Analyses of the effects of parental and offspring environments around the weight median (Table 1)

1. Pre- weight median

| Shell length |  | **Estimates (SE)** | **Numdf, Dendf** | **F** | **P** |
| --- | --- | --- | --- | --- | --- |
|  | Weight | 1.5021 (0.0303) | 1, 182.97 | 2457.40 | <0.0001* |
|  | Parental env. (G1) | -0.0181 (0.0134) | 1, 28.91 | 1.82 | 0.1872 |
|  | Offspring env. (G2) | -0.0172 (0.0119) | 1, 182.94 | 2.07 | 0.1514 |
|  | G1 x G2 | -0.0154 (0.0119) | 1, 182.95 | 1.65 | 0.1998 |
|  | *Random effect* | ***Var*** | ***SE*** | ***Z*** | ***P*** |
|  | *Family* | *0.00117* | *0.0014273* | *0.82* | *0.2061* |
|  |  |  |  |  |  |
| Shell width |  | **Estimates (SE)** | **Numdf, Dendf** | **F** | **P** |
|  | Weight | 0.7653 (0.0179) | 1, 179.5 | 1823.80 | <0.0001* |
|  | Parental env. (G1) | -0.0083 (0.0098) | 1, 33.31 | 0.72 | 0.4033 |
|  | Offspring env. (G2) | -0.0147 (0.0071) | 1, 180.7 | 4.27 | 0.0401** |
|  | G1 x G2 | 0.0089 (0.0071) | 1, 180.5 | 1.60 | 0.2078 |
|  | *Random effect* | ***Var*** | ***SE*** | ***Z*** | ***P*** |
|  | *Family* | *0.0015587* | *0.0007591* | *2.05* | ***0.02*** |
|  |  |  |  |  |  |

1. Post- weight median

| Shell length |  | **Estimates (SE)** | **Numdf, Dendf** | **F** | **P** |
| --- | --- | --- | --- | --- | --- |
|  | Weight | 2.0907 (0.0622) | 1, 177.8 | 1130,46 | <0.0001* |
|  | Parental env. (G1) | -0.0202 (0.0228) | 1, 38.69 | 0.78 | 0.3815 |
|  | Offspring env. (G2) | -0.0334 (0.0151) | 1, 168.8 | 4.91 | 0.0281* |
|  | G1 x G2 | 0.0318 (0.0151) | 1, 167.7 | 4.49 | 0.0355* |
|  | *Random effect* | ***Var*** | ***SE*** | ***Z*** | ***P*** |
|  | *Family* | *0.0091358* | *0.0038653* | *2.36* | *0.009* |
|  |  |  |  |  |  |
| Shell width |  | **Estimates (SE)** | **Numdf, Dendf** | **F** | **P** |
|  | Weight | 1.1564 (0.0334) | 1, 177.9 | 1157.49 | <0.0001* |
|  | Parental env. (G1) | -0.0046 (0.0122) | 1, 31.87 | 0.14 | 0.7069 |
|  | Offspring env. (G2) | -0.0259 (0.0082) | 1, 166.7 | 9.83 | 0.0020* |
|  | G1 x G2 | 0.0246 (0.0082) | 1, 165.4 | 8.95 | 0.0032* |
|  | *Random effect* | ***Var*** | ***SE*** | ***Z*** | ***P*** |
|  | *Family* | *0.0025* | *0.0012* | *2.08* | *0.0186** |
|  |  |  |  |  |  |

* symbol indicates P < 0.05.
